# Colonoscopy Quality and Adherence to Postpolypectomy Surveillance Guidelines in an Underinsured Clinic System

**DOI:** 10.1155/2020/6240687

**Published:** 2020-10-31

**Authors:** Jaison John, Abdul Al-Douri, Bretta Candelaria, Saurin Gandhi, Paul Guzik, Brent Herndon, Christopher Kim, Nicole Kluz, Jennifer Thompson, Jessica Trevino, Victoria Valencia, Michael Pignone

**Affiliations:** ^1^Department of Internal Medicine, The University of Texas at Austin Dell Medical School, Austin, TX, USA; ^2^Department of Population Health, The University of Texas at Austin Dell Medical School, Austin, TX, USA; ^3^LIVESTRONG Cancer Institutes, The University of Texas at Austin Dell Medical School, Austin, TX, USA

## Abstract

**Background:**

Delivery of high-quality colonoscopy and adherence to evidence-based surveillance guidelines is essential to a high-quality screening program, especially in safety net systems with limited resources. We sought to assess colonoscopy quality and ensure appropriate surveillance in a network of safety net practices.

**Methods:**

We identified age-eligible patients ages 50-75 within a Federally Qualified Health Center (FQHC) clinic system with evidence of colonoscopy in preceding 10 years. We performed chart reviews to assess key aspects of colonoscopy quality: bowel preparation quality, evidence of cecal intubation, cecal withdrawal time, and the adenoma detection rate. We then utilized established guidelines to assess and revise surveillance colonoscopy intervals, determine whether appropriate surveillance had taken place, and schedule overdue patients as appropriate.

**Results:**

Of 26,394 age-eligible patients, a total of 3,970 patients had evidence of prior colonoscopy and 1,709 charts were selected and reviewed. Mean age was 57, 54% identified as women and 51% identified as Hispanic. Of 1709 colonoscopies reviewed, 77% had data on bowel preparation, and of those, 85% had adequate preparation quality. Cecal intubation was documented in 89% of procedures. Adequate cecal withdrawal time was documented in 59% of those with documented cecal intubation. Overall adenoma detection rate was 42%. Initial surveillance interval was clearly stated in 72% (*n* = 1238) of procedures. Of these, initial recommended intervals were too short in 24.5% (*n* = 304) and too long in 3.6% (*n* = 45). A total of 132 patients (10.7%) were overdue for appropriate surveillance and were referred for follow-up colonoscopy.

**Conclusions:**

Overall, the quality of screening colonoscopy was high, but reporting was incomplete. We found fair adherence to evidence-based surveillance guidelines, with significant opportunities to extend surveillance intervals and improve adherence to best practices.

## 1. Background

Despite strong evidence supporting the value of screening and surveillance, colorectal cancer (CRC) remains the second leading cause of cancer death in the US [1]. Vulnerable patients, including those served by safety net systems, are less likely to receive appropriate screening and surveillance and are at an increased risk of mortality [2–4]. High-quality colonoscopy is an important element of an effective colorectal cancer screening and surveillance program; however, access to high-quality colonoscopy may be especially challenging in safety net settings where many patients have limited or no health insurance. In such settings, it is critical to ensure and document that patients are receiving high-quality surveillance at proper intervals, as inadequate surveillance can increase CRC incidence and mortality, while oversurveillance represents waste, and limits access to those who are appropriately due for testing.

Guidelines are available to help health systems utilize colonoscopy resources effectively and efficiently. In 2012, the United States Multi-Society Task Force (USMSTF) released an updated evidence-based guideline for surveillance after initial screening colonoscopy based on colonoscopy quality and findings [5]. A high-quality colonoscopy ensures screening efficacy by detection and removal of neoplastic lesions [6].

Several quality indicators are in place to ensure high-quality colonoscopy, including bowel preparation quality, cecal intubation and withdrawal time, and adenoma detection rate (i.e., the fraction of patients undergoing colonoscopy who had one or more confirmed adenomas detected and removed on their exam) [5]. Bowel preparation quality is considered adequate if it allows for the visual detection of polyps > 5 mm in size [7]. Demonstration of cecal intubation and adequate withdrawal time (defined as the duration in minutes, it takes to withdraw the endoscope through the colon after initially intubating the cecum) allows for careful examination of the right side of the colon and is useful in the prevention of interval proximal colon cancer [6–8]. A withdrawal time of 6 minutes or longer is associated with greater rates of adenoma detection [9, 10]. Adenoma detection rates below 20% were associated with an increased risk of interval colorectal cancer, and hence, current gastroenterology society guidelines recommend an adenoma detection rate of ≥25% for screening colonoscopies [6, 7].

Monitoring endoscopy quality is key to an effective colorectal cancer prevention program, but many safety net institutions have not had sufficient resources to ensure quality examinations. In this work, we sought to examine the quality of reporting and quality of colonoscopy practice within our safety net Federally Qualified Health Center (FQHC) system as part of a larger quality improvement effort for colorectal cancer screening and prevention. We then used this data to develop better systems and policies to ensure more effective and efficient resource utilization.

## 2. Methods

### 2.1. Overview

Using established guidelines [5–7], we aimed to measure important quality characteristics of colonoscopy, identify provider and patient factors associated with these measures, and compare initial provider-recommended surveillance intervals to revised guideline-based intervals in a Federally Qualified Health Center (FQHC) clinic system in Austin, Texas.

This project was assessed and considered exempt by the University of Texas at Austin Dell Medical School Institutional Review Board.

### 2.2. Cohort Identification

In late 2017, we identified 26,394 age-eligible (50-75 years old) patients for colorectal cancer screening within an FQHC clinic system in Austin, Texas. To identify current patients, we filtered medical records and identified patients who visited the clinic at least once in the past 12 months or twice in the preceding 24 months. We then combined data from distinct outpatient and inpatient electronic health record (EHR) systems to identify patients with evidence of colonoscopy within the past 10 years (January 2008-November 2017). Inpatient and outpatient data were combined using a matching algorithm followed by manual review. Our targeted sample size was approximately 1500 patient charts to allow for the identification of key outcomes based on available resources, particularly personnel to conduct chart reviews. We used the most recent colonoscopy for each patient as the index procedure for our analyses.

### 2.3. Chart Review

After eligible patients were identified, a member of a group of six trained internal medicine resident reviewers performed chart review utilizing data from both electronic health records. The reviewer entered data into a secure structured form utilizing REDCap™. Data collected with the reviews included patient demographics, bowel preparation quality, evidence of cecal intubation, and cecal withdrawal time. The number and size of polyps with pertinent pathology, family history of colorectal cancer, and initial provider recommended surveillance follow up interval were also recorded. Reviewers evaluated procedure notes, provider-added pathology reports, and post procedure GI clinic visits to document the initial provider-recommended surveillance interval.

We categorized bowel preparations graded as “excellent” or “good” to be adequate and those graded as “fair” or “poor” as inadequate [7]. Evidence for intubation of the cecum was also captured, and we categorized a colonoscopy as having adequate withdrawal time if withdrawal time was 6 minutes or greater.

### 2.4. Analyses

We used descriptive statistics to describe the population and colonoscopy characteristics. We examined quality indicators based on several demographic characteristics: age (dichotomized at the median age), sex, race, ethnicity, and preferred language.

Adenoma detection rates were also calculated for individual providers and analyzed. We used consensus USMSTF guidelines for colonoscopy surveillance intervals to assess the accuracy of recommended surveillance interval after the index examination [5]. Index high-quality colonoscopies with no adenomas detected were assigned 10-year follow up intervals. If precancerous lesions were detected, they were classified as low-risk adenomas (LRAs) or high-risk adenomas (HRAs). LRAs were defined as 1-2 tubular adenomas < 10 mm and were assigned a 5-10-year follow-up interval. HRAs were defined as adenoma with villous histology, high-grade dysplasia (HGD), ≥10 mm, or 3 or more adenomas and were assigned a 3-year follow-up interval. Small sessile serrated adenomas < 10 mm without evidence of dysplasia were assigned a 5-year follow-up interval. Sessile serrated adenomas that were large ≥10 mm, had evidence of dysplasia, or were traditional serrated adenomas were assigned a 3-year follow-up interval. Those with inadequate bowel preparation quality were assigned a 1-year follow-up interval [5–11]. Remaining surveillance intervals were assessed based on pathology findings, number of polyps detected, and family history [5]. The reviewers utilized these USMSTF guidelines to create a revised surveillance interval for comparison of the recommended interval from the initial provider.

Inter-rater reliability for surveillance interval was assessed between the most frequent reviewers by performing an independent second review of a random subset of 50 patient charts for four of the six resident reviewers.

All statistical analyses were completed using R version 3.6.0 (Foundation for Statistical Computing).

### 2.5. Follow-Up

Patients were assigned recommended USMSTF surveillance intervals, and those overdue for surveillance were contacted to have a follow-up procedure performed.

## 3. Results

Among 26,394 age-eligible patients, 6,036 (23%) were up to date on their colorectal cancer screening. Of these, 3,970 had evidence of a colonoscopy and the first 1,709 patient charts were selected and reviewed.

### 3.1. Demographics

Table 1 shows the demographic characteristics of the patients included. Mean age was 57, median age was 58, and the majority were women (54%). Most patients were Hispanic (51%), preferred English (57%), and were underinsured (60%).

### 3.2. Quality Measures

#### 3.2.1. Bowel Preparation Quality

Bowel preparation quality data was available for 77% (1322/1709) of patients. Of these 1322, preparation quality was adequate in 85%.  Table 2 summarizes demographic characteristics for patients with adequate vs. inadequate bowel preparation.

#### 3.2.2. Cecal Intubation Rate

There was documented presence or absence of cecal intubation in 89% (*n* = 1513) of procedures reviewed, and the rate of documented intubation was 98%. There were no significant differences in cecal intubation by age, race, ethnicity, or language (data not shown). Men were somewhat more likely to have adequate withdrawal times compared to women (90% vs. 83%, *p* = 0.005) (Table 3). For those with documentation of preparation adequacy, we found no differences in evidence of cecal intubation between those with adequate (71%) versus inadequate (72%) bowel preparation.

#### 3.2.3. Cecal Withdrawal Time

Cecal withdrawal time was documented in 853 (50%) of procedures reviewed. Of these 853 procedures, cecal withdrawal time was adequate (≥6 minutes), in 86% of patients (*n* = 735). The average cecal withdrawal time was 7.4 minutes in patients with normal findings (*n* = 863), 9.8 minutes in patients with hyperplastic polyps (*n* = 56), 10.2 minutes in patients with adenomas (*n* = 703), and 12.7 minutes in patients with cancer (*n* = 11).

#### 3.2.4. Adenoma Detection Rate

Overall adenoma detection rate for the 1709 colonoscopies performed was 42%. Two providers accounted for 65% (*n* = 1117) of procedures, with 83 other providers accounting for the remaining 591 procedures. These two busiest providers had higher adenoma detection rates, at 56% and 45%, than the other 83 providers combined (29% overall adenoma detection rate). The two busiest providers were faculty gastroenterologists practicing primarily in the university hospital setting, while the other providers included a mix of private practice gastroenterologists and surgeons.

Older patients (>age 58) had a higher rate of adenoma detection, than patients ≤ 58 years old (50% vs. 40%, *p* < 0.001). Men also had a higher adenoma detection rate than women (48% vs 40%, *p* = 0.001) (Table 4). 43% of patients were found to have adenomas (*n* = 703), and 0.67% were found to have cancer (*n* = 11).

### 3.3. Surveillance Intervals

Initial surveillance interval was clearly stated in 72% (*n* = 1238) of procedures. There was agreement between initial and reviewer revised surveillance intervals in 72% of these procedures (*n* = 889). Initial recommended intervals were too short in 24.5% (*n* = 304), and too long in only 3.6% (*n* = 45).  Table 5 summarizes the differences between the initial provider-recommended interval and the revised surveillance intervals based on the review. Inter-rater agreement was 70% between the two sets of reviewers (see Appendix for data (available here)).

### 3.4. Improving Surveillance Adherence

Of the 1238 patients whose surveillance interval could be assessed, we found that 132 (10.7%) were overdue for surveillance from chart review. We sought to reach out to these 132 to confirm overdue status and schedule them for colonoscopy. After outreach, 29 of 132 reported recent outside screening, 7 preferred to schedule the procedure with their own provider, and 48 were unable to be contacted. Of the remaining 48, 11 refused colonoscopy or were unable to schedule due to scheduling conflicts, 17 did not attend the pre-procedure evaluation appointment, and 3 patients are currently scheduled for the pre-procedure evaluation. We were able to successfully schedule 17 patients (29.6%) for colonoscopy, of whom 11 have completed colonoscopy as of April 2019. Of the 11 patients who completed a colonoscopy, 55% (*n* = 6) had polyps. Of those with polyps, two patients had 2 small tubular adenomas, one patient had 4 small tubular adenomas, one patient had 20 tubular adenomas, and another patient had one tubular adenoma and two sessile serrated adenomas identified. Another patient had 3 small tubular adenomas, but unfortunately suffered a bowel perforation and underwent a successful rectosigmoid repair.

## 4. Discussion

In our initiative to understand and improve the quality of colonoscopy within our safety net system, we found overall documented colonoscopy quality to be high, but many data elements were incompletely reported. Adenoma detection, a hallmark of high-quality colonoscopy, was higher for providers who performed the majority of procedures. There were no consistent differences in colonoscopy quality by patient demographic groups, suggesting that the care delivered within this system was not increasing health disparities.

We evaluated colonoscopy quality based on bowel preparation quality, cecal intubation, and adenoma detection rate. Performance targets set by US gastroenterology societies recommend adequate bowel preparation for 85% of all examinations, the level observed in our study [7]. For procedures with documented cecal intubation, the rate of intubation was 98%, above the minimum recommended 90% [7]. The target adenoma detection rate for surveillance colonoscopy is typically higher than for screening colonoscopy [12]. Adenoma detection rates have been reported as high as 55% to 70% in patients undergoing screening colonoscopy after an abnormal Fecal Immunochemical Test [13, 14]. Our overall 42% adenoma detection rate is high, but it is unclear what our target adenoma detection rate should be given the mixture of procedure indications and incomplete reporting of such indications. Although our two busiest providers had high adenoma detection rates, the heterogeneity of the remaining 83 providers with a combined adenoma detection rate of 29% suggests variability in colonoscopy quality and requires additional investigation.

Using consensus guidelines to evaluate recommended surveillance intervals, we found moderate differences between initial provider and evidence-based reviewer recommendations for surveillance intervals, with providers often recommending inappropriately short intervals. As such, we were able to extend follow-up intervals for almost a quarter of patients, thus sparing patient over-surveillance and opening up the potential for re-deployment of endoscopy resources. We were also able to identify patients who were overdue for surveillance and reach out to them to engage them in care.

Previous studies have also documented overuse of colonoscopy through the use of inappropriately short surveillance intervals. One study based on US Veterans in 2015 found that nearly a third of endoscopists recommended an interval that was shorter than that delineated by current guidelines [15]. Both endoscopists and primary care providers tended to shorten surveillance intervals without adhering to postpolypectomy surveillance guidelines in clinical practice and when surveyed [16–30]. Our findings extend this work in a safety net population with significant challenges in care continuity and access.

Limitations of our study include our use of a convenience sample of charts for review and routine assessment by a single reviewer. However, we did conduct second reviews of a sample of charts and found good, but not perfect, inter-rater reliability. There was a considerable amount of missing documentation, which varied by provider and date of exam, with more recent procedures better documented. We included colonoscopies for review regardless of the stated indication, and our data regarding procedure indication was incomplete due to missing documentation. It is possible that procedures done for different indications could produce different levels of quality, but these indications were not sufficiently well-recorded for analysis. Additionally, updated 2020 USMSTF surveillance colonoscopy guidelines were released after our review but were not utilized as the procedures were performed during the time period covered by prior guidelines [31].

Based on our findings, we have undertaken several steps to improve quality. First, we now have a system to identify patients overdue for colonoscopy, reach out to them to confirm their overdue status, and invite them to a pre-endoscopy appointment. We also intend to further improve quality reporting within our system by adopting uniform endoscopy reporting software and developing a group consensus for colonoscopy documentation and reporting. Additionally, we plan to implement a peer review process to ensure quality of examinations. We anticipate a change in our outpatient clinics' EHR, which may help providers navigate colonoscopy surveillance more easily, but for now, we will maintain a separate surveillance database with information from both systems. Each of these interventions should help better utilize colonoscopy resources, help reduce disease burden, and improve access to care.

## Figures and Tables

**Table 1:** Characteristics of patients from chart reviews (*n* = 1709).

Characteristic	Value
Age in years, mean (SD)	57.2 (5.7)
Age range in years	45-75
Gender, *n* (%)	
Female	915 (54)
Race, *n* (%)	
Black	267 (16)
White	1060 (62)
Other	108 (6)
Refused/unknown	274 (16)
Ethnicity, *n* (%)	
Hispanic	869 (51)
Non-Hispanic	784 (46)
Refused/unknown	56 (3)
Preferred language, *n* (%)	
English	981 (57)
Spanish	597 (35)
Other	75 (4)
Refused/unknown	56 (3)
Insurance, *n* (%)	
Commercial	171 (10)
Medicare	323 (19)
Medicaid	188 (11)
County-based medical assistance program	736 (43)
Uninsured or clinic-based medical assistance program	291 (17)

**Table 2:** Comparison of inadequate bowel prep (fair/poor) across patient demographic characteristics (*n* = 1322).

Characteristic	*n* (%)	*p* value
Age		
≤58	119 (16)	0.63
>58	84 (15)	
Gender		
Male	122 (21)	<0.001
Female	81 (11)	
Race		
White	123 (15)	0.008
Black	42 (23)	
Ethnicity		
Non-Hispanic	103 (19)	0.01
Hispanic	95 (13)	
Language		
English	130 (19)	0.001
Spanish	60 (12)	

**Table 3:** Comparison of adequate cecal withdrawal time across patient demographic characteristics (*n* = 896).

Characteristic	*n* (%)	*p* value
Age		
≤58	415 (87)	0.343
>58	320 (85)	
Gender		
Male	316 (90)	0.005
Female	420 (83)	
Race		
White	492 (87)	0.496
Black	79 (84)	
Ethnicity		
Non-Hispanic	262 (87)	0.888
Hispanic	443 (86)	
Language		
English	361 (88)	0.296
Spanish	314 (85)	

**Table 4:** Comparison of adenoma detection rate across patient demographic characteristics (*n* = 1709).

Characteristic	*n* (%)	*p* value
Age		
≤58	375 (40)	<0.001
>58	338 (50)	
Gender		
Male	360 (48)	0.001
Female	354 (40)	
Race		
White	466 (46)	0.11
Black	102 (40)	
Ethnicity		
Non-Hispanic	319 (43)	0.86
Hispanic	365 (44)	
Language		
English	420 (46)	0.18
Spanish	244 (42)	

**Table 5:** Revised surveillance intervals based on USMSTF guidelines (*n* = 1238).

Initial versus revised surveillance intervals						
Initial interval	USMSTF recommended interval					
	1 year	3 years	5 years	5-10 years	10 years	Total
1 year	16	4	0	2	1	23
3 years	7	165	12	17	3	204
5 years	1	27	223	146	103	500
5-10 years	0	0	1	8	16	25
10 years	1	0	7	1	477	486
Total	25	196	243	174	600	1238

Total in agreement 889/1238 (72%).

## Data Availability

The authors confirm that the data supporting the findings of this study are available within the article and its supplementary materials.

## Supplementary Materials

Supplementary Materials Appendix 1: Inter-rater reliability (*n* = 99).
