# Supplementary material for: Colonoscopy Quality and Adherence to Postpolypectomy Surveillance Guidelines in an Underinsured Clinic System
Source: Gastroenterol Res Pract. 2020 Oct 31;2020:6240687. doi: 10.1155/2020/6240687 (PMC7648690; doi:10.1155/2020/6240687)
Supplement: Supplementary Materials — Appendix 1: Inter-rater reliability (n = 99). [file 6240687.f1.pdf]

Appendix

Appendix 1. Inter-rater reliability (n=99)

Inter-Rater Reliability

**Initial Reviewer**	**Second Reviewer**							
	Repeat	1 year	3 years	5 years	5-10 years	10 years	Unable to Assess	Missing
Repeat	1							
1 year		2						
3 years			4	1				
5 years		1		8	2		1	
5-10 years			1	15	9		1	
10 years				4		41	1	
Unable to Assess		1					1	
Missing		1			1			3

Total in Agreement 69/99 (70%)
